# Improved detection of sepsis

**DOI:** 10.1186/1757-7241-20-S2-P27

**Published:** 2012-04-16

**Authors:** Jane Bach Nielsen, Anne Dahl Rasmussen

**Affiliations:** 1 Orthopeadic Trauma Unit, Aarhus University Hospital, Denmark; 2 Orthopeadic Trauma Unit, Aarhus University Hospital, Denmark

## Background

We are nurses at Aarhus University Hospital’s Orthopaedic Trauma Unit. In connection with training in emergency medicine for nurses, we have completed a quality development project in the department, which deals with the early detection of sepsis. The project stems from the training we received on sepsis. We became curious about our practice in the department. Our hypothesis is that our department’s nursing staff lacks knowledge about SIRS and sepsis, in regard to being able to observe, examine and treat this patient group optimally.

## Methods

The methods used in our project were questionnaire and journal audit.

## Results

Based on the results from the questionnaires, it is assessed that there is a lack of knowledge regarding SIRS and sepsis among the nursing staff. The journal audit likewise indicates that the lack of knowledge about SIRS and sepsis results in a lack of focus on sepsis in practice. The survey shows that neither the nursing staff nor doctors consider SIRS or sepsis when a patient has abnormal vital parameters. Therefore an investigation of the infection focus is rarely launched – resulting in sepsis not being recognized or disproved during hospitalization.

## Conclusion

It is assessed that quality could eventually be improved with the implementation of various initiatives such as training for the staff in the ABC-assessment principles and sepsis, and the development of internal interdisciplinary clinical guidelines regarding SIRS and sepsis, focusing primarily on management options. Another example of a quality improvement initiative could be the development of a pocket card which would ensure that the staff had easy access to information about SIRS and sepsis in emergency situations.

